# Comparative analysis of human and mouse immunoglobulin variable heavy regions from IMGT/LIGM-DB with IMGT/HighV-QUEST

**DOI:** 10.1186/1742-4682-11-30

**Published:** 2014-07-03

**Authors:** Bin Shi, Long Ma, Xiaoyan He, Xiaomei Wang, Peng Wang, Li Zhou, Xinsheng Yao

**Affiliations:** 1 Department of Immunology, Research Center for Medicine & Biology, Innovation & Practice Base for Graduate Students Education, Zunyi Medical University, Zunyi 563000, China

**Keywords:** Immunoglobulin, VH CDR3, IMGT/HighV-QUEST, IMGT/LIGM-DB, VH repertoire

## Abstract

**Background:**

Immunoglobulin (IG) complementarity determining region (CDR) includes VH CDR1, VH CDR2, VH CDR3, VL CDR1, VL CDR2 and VL CDR3. Of these, VH CDR3 plays a dominant role in recognizing and binding antigens. Three major mechanisms are involved in the formation of the VH repertoire: germline gene rearrangement, junctional diversity and somatic hypermutation. Features of the generation mechanisms of VH repertoire in humans and mice share similarities while VH CDR3 amino acid (AA) composition differs. Previous studies have mainly focused on germline gene rearrangement and the composition and structure of the CDR3 AA in humans and mice. However the number of AA changes due to somatic hypermutation and analysis of the junctional mechanism have been ignored.

**Methods:**

Here we analyzed 9,340 human and 6,657 murine unique productive sequences of immunoglobulin (IG) variable heavy (VH) domains derived from IMGT/LIGM-DB database to understand how VH CDR3 AA compositions significantly differed between human and mouse. These sequences were identified and analyzed by IMGT/HighV-QUEST (http://www.imgt.org), including gene usage, number of AA changes due to somatic hypermutation, AA length distribution of VH CDR3, AA composition, and junctional diversity.

**Results:**

Analyses of human and murine IG repertoires showed significant differences. A higher number of AA changes due to somatic hypermutation and more abundant N-region addition were found in human compared to mouse, which might be an important factor leading to differences in VH CDR3 amino acid composition.

**Conclusions:**

These findings are a benchmark for understanding VH repertoires and can be used to characterize the VH repertoire during immune responses. The study will allow standardized comparison for high throughput results obtained by IMGT/HighV-QUEST, the reference portal for NGS repertoire.

## Background

Immunoglobulin (IG) protects against the invasion of pathogenic microorganisms and is an important effector molecule in the immune response. IG is a tetramer composed of two heavy (H) chains and two light (L) chains (Kappa or Lambda). The variable domain at the N-terminal end of each chain is generated by the rearrangement of a variable (V) gene, a diversity (D) gene (for the VH) and a joining (J) gene. The IG genes are located in different loci, IGH, IGK and IGL on chromosomes 14, 2 and 22, respectively [1,2] (IMGT Repertoire in IMGT®, the international ImMunoGeneTics information system® [3,4], http://www.imgt.org). In addition, somatic hypermutation (SHM) contributes to the diversity of the IG V domain [5]. The third complementarity-determining region of the IG heavy chain (VH CDR3) functions as the center of the classical antigen-binding site and it often influences how the specificity and affinity of the antibody is determined [6-9]. Due to its key role in antigen binding, the VH CDR3 has become a target for the introduction of hypervariability in synthetic antibody libraries [10,11], which can be used for engineering therapeutic antibodies [12]. In the body, VH CDR3 is more diverse than any of the other five CDRs [13,14], which is generated by deletion and insertion of random nucleotides (nts) during joining (junctional diversity) [15].

Immunoinformatics tools have been developed for the detailed analysis of the V domains taking into account the complex mechanism of their synthesis [4]. Thus IMGT/V-QUEST [16-18] provides the identification of the closest V and J germline genes involved in the rearrangement, analysis of the somatic mutations and AA changes, and with the integrated IMGT/JunctionAnalysis [19,20], a detailed characterization of the junction and identification of the D genes. The online version of IMGT/V-QUEST analyses 1 to 50 sequences per run. A high throughput version, IMGT/HighV-QUEST [17,21,22], analyzes up to 500,000 IG or T cell receptors (TR) sequences. High quality results are based on the standardized concepts of IMGT-ONTOLOGY [23], that are generated from the axioms of CLASSIFICATION (standardized nomenclature) [24], DESCRIPTION (standardized labels) [25], NUMEROTATION (IMGT unique numbering [26-28], IMGT Collier de Perles [29]). IMGT/HighV-QUEST results are identical to those obtained by IMGT/V-QUEST online, except for the IMGT Collier de Perles.

Generally, human and mouse are considered as the most developed models for the generation of IG diversity [30]. Previous studies have shown that human and murine VH CDR3 repertoires share similarities, i.e., tyrosine, glycine and serine tend to predominate in their amino acid (AA) composition [31,32] and the length distribution of CDR3 is nearly normally distributed [32]. However, they significantly differ regarding AA composition, including VH CDR3 of equal length [32]. Features of the generation mechanisms of VH CDR3 repertoire in humans and mice share similarities while their AA composition differs. Why AA composition of VH CDR3 differs between human and mouse is unclear. Previous studies have focused on the germline gene rearrangement. However, number of AA changes due to somatic mutations and junctional diversity have been largely ignored. Comparing these mechanisms between human and mouse will clarify the nature of the VH CDR3 repertoire. Moreover, to efficiently produce more realistic synthetic antibody libraries, it is important to understand the rules and restrictions that act upon the composition of the VH CDR3 region.

Over the past few years, large collections of sequences have been deposited in the IMGT/LIGM-DB database [33,34]. From this database, 10,507 human sequences and 8,362 murine sequences were queried. However, such a large amount of data is a challenge for the subsequent comparative analysis of human and mouse IG VHs. Recently, IMGT/HighV-QUEST is widely applied in analysis of NGS data of IG or TR (T cell receptor) repertoire. Based on IMGT/HighV-QUEST results, we identified and compared 9,340 human and 6,657 murine unique, productive and in-frame sequences of IG VH from IMGT/LIGM-DB, including germline gene usage, number of AA changes due to somatic mutations, CDR3 length distribution and AA composition, and junctional diversity.

## Results

The analyzed sequences were derived from different laboratories. Generally, different laboratories measured different lengths according to their research imperatives. Therefore, we only retained those sequences that contained a complete V region. Although sequences were considered effective because that came from the IMGT/LIGM-DB, we still removed some deemed invalid by IMGT/HighV-QUEST. After eliminating all redundant, ambiguous, incomplete, or unproductive sequences from the sequences contained within the IMGT/LIGM-DB, we identified 9,340 human and 6,657 murine unique productive Ig V-region sequences.

### Germline gene usage

Both human and murine gene usages were shown in Additional file 1: Figure S1A-H. For the human IGHV gene subgroup, our data covered 52 (52/71) IGHV genes. Of these, IGHV1-69 (7.7%), IGHV5-51 (6.8%), IGHV4-34 (7.5%), IGHV3-30 (6.9%) and IGHV3-23 (7.7%) were more used, while IGHV5-78, IGHV1-45, IGHV4-28, IGHV3-43D and IGHV3-NL1 were used less than 0.1%. In IGHD and IGHJ gene set, IGHD3-10 at 11.8% and IGHJ4 at 44.1% were the most favored genes. For the murine IGHV gene subgroup, our data covered 130 (130/130) IGHV genes. Surprisingly, the frequency of IGHV1-72 at 43.1% was significantly higher than any other genes. Similarly, in the IGHD gene set, IGHD1-1 at 39.2% was predominately used. In the IGHJ gene family, IGHJ4 were in highest usage at 41.8%. In human and murine sequences, sequences without the D gene were 0.7% and 3.7%, respectively.

### Analysis of the number of AA changes due to somatic hypermutation

**Figure 1:**
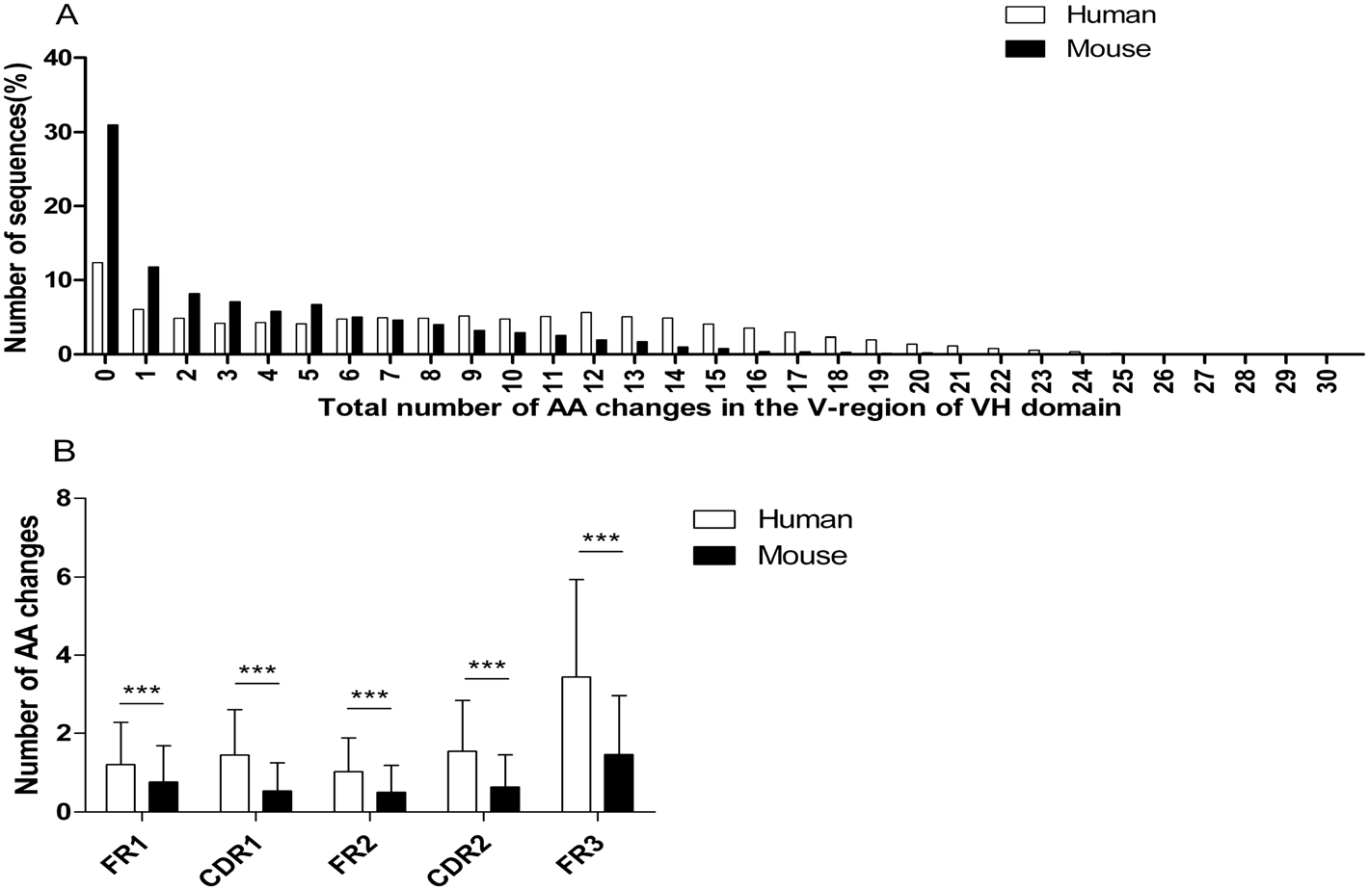
**Number of amino acid (AA) changes of all human (n = 9340) and mouse (n = 6657) sequences is shown. A** Total number of AA changes in the V regions of VH domain. **B** The number of AA changes observed in FR1, CDR1, FR2, CDR2, and FR3 of VH domain, with the mean ± SD. The p values were determined using ANOVA. All statistically significant differences are indicated. * = p < 0.05, ** = p < 0.01, *** = p < 0.001.

Numbers of V region AA changes were compared between human and murine sequences (Figure 1A-B), but we excluded mutations at the CDR3 and FR4 because of their involvement in the rearrangement process. As shown in Figure 1A, the numbers of sequences decrease with increasing the numbers of AA changes in both human and mouse, but sequences without AA changes were found more frequently in mouse than in human (31.0% versus 12.3%). A higher sequence frequency occurred in mouse than in human (70.6% versus 35.7%) when the number of AA changes ranged from 0 to 5. If the number of AA changes ranged from 6 to 8, the human sequence frequency was roughly identical to that of the murine sequence (14.4% and 13.6%, respectively). When the range of the number of AA changes exceeded 9, then a higher sequence frequency occurred in human than in mouse (49.9% versus 15.8%). Moreover, human sequences had higher average numbers of AA changes in FR1, CDR1, FR2, CDR2 and FR3 compared to murine sequences (each p < 0.001, ANOVA; Figure 1B).

### CDR3 length distribution and AA composition

#### Length distribution of VH CDR3

VH CDR3 length in human ranged from 4 to 36 according to the IMGT numbering and it was wider than in mouse, which ranged from 4 to 28 (Additional file 1: Figure S2). The average length of VH CDR3 in human (15.5 ± 3.2 AA residues) was longer than in mouse (11.5 ± 1.9 AA residues). The most frequently occurring length in human was 14 (11.7%) whereas mouse had three fewer AA residues at 11 (19.8%) (Additional file 1: Figure S2).

#### Overall AA usage of VH CDR3

**Figure 2:**
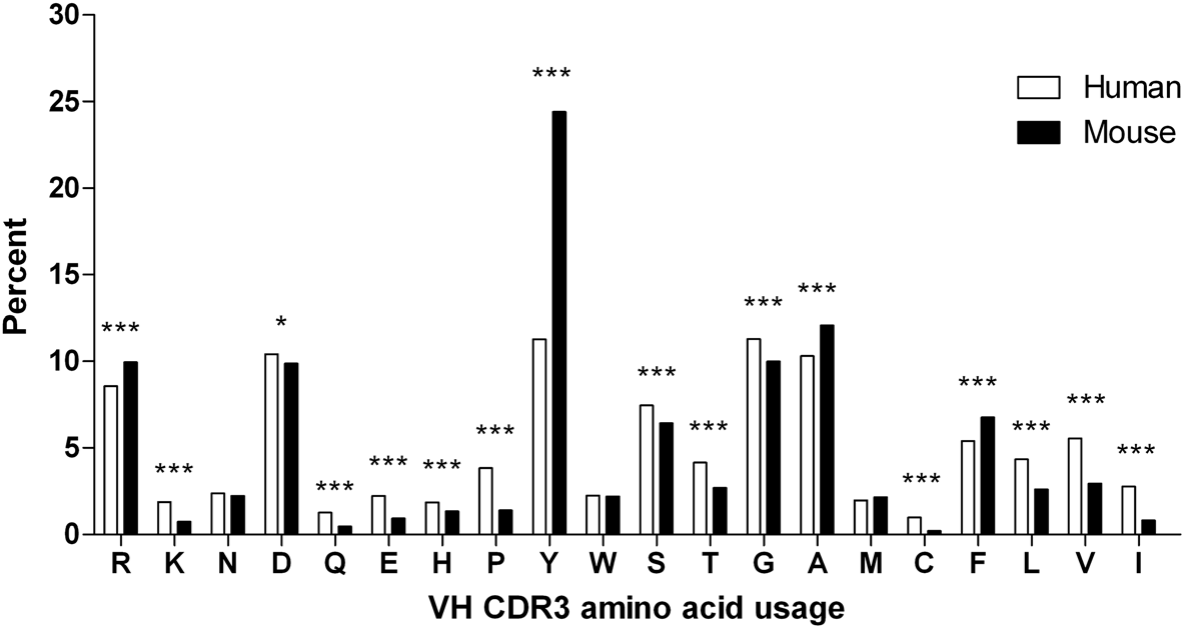
**Average values of AA composition calculated for all human (n = 9340) and mouse (n = 6657) VH CDR3 sequences in percent shows the difference from overall amino acid composition in human and murine.** The amino acids are arranged by relative hydrophobicity values as found in Kyte-Doolittle scale [35]. The overall amino acid composition differed significantly between human and murine VH CDR3 ( χ^2^ test, 19 degrees of freedom, p < 0.001). Comparing the frequencies of individual amino acid residues, the definite level of significance was better than *0.05%, **0.001%, or ***0.00001% using χ^2^ test and post-hoc analysis as described by Collis et al. [36].

Overall AA usages of human and murine VH CDR3 are shown in Figure 2. The amino acids most frequently used in human and mouse are tyrosine, alanine, aspartic acid, arginine, glycine and serine. Significant differences in overall AA composition between human and mouse exist (χ2 test, 19 degrees of freedom, p < 0.001). Obviously, the usage frequency of tyrosine in mouse was higher than in human (24.4% versus 11.3%, p < 0.00001%, χ2 test). Usage frequencies of lysine, glutamine, glutamic acid, histidine, proline, serine, threonine, glycine, cysteine, leucine, valine and isoleucine in human was higher than in mouse (each p < 0.00001%, χ2 test) and the murine model showed higher utilization of arginine, alanine and phenylalanine compared to the human model (each p < 0.00001%, χ2 test). Over-utilization of tyrosine led to a strong bias in the overall AA composition of murine VH CDR3, while human VH CDR3 showed a more balanced overall AA composition.

#### Overall AA usage of VH CDR3 at different positions

**Figure 3:**
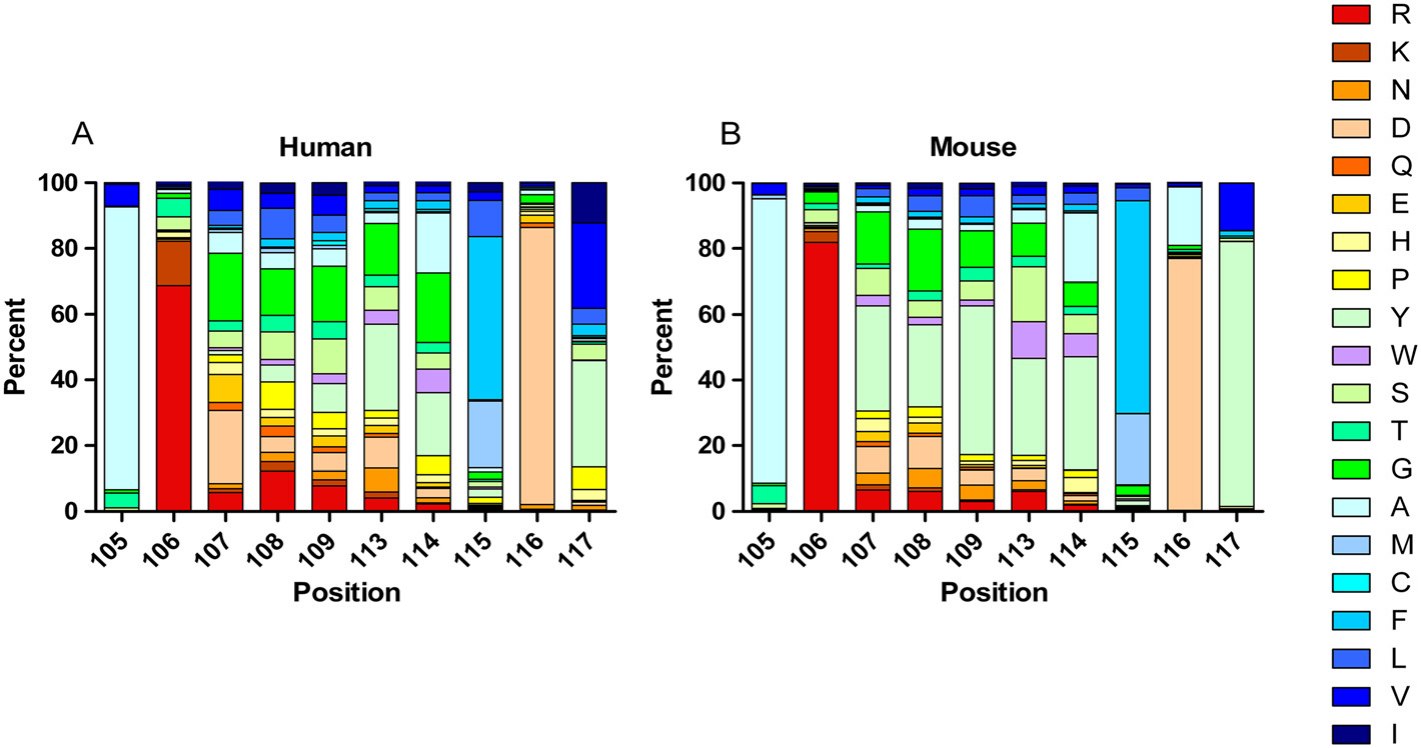
**The frequency of individual amino acids at the specific ten positions for all human (n = 9340) and mouse (n = 6657) VH CDR3 sequences is shown.** The color menu for amino acids is according to IMGT [37]. CDR3 positions are shown according to the IMGT unique numbering. **A** human. **B** mouse.

According to IMGT numbering, we identified all positions 105–117 of VH CDR3 sequences and calculated overall AA frequencies for all positions of CDR3 sequences (Figure 3 shows 10 positions, but other positions are not shown). At the N-terminal of VH CDR3, both human and murine sequences at position 105 frequently utilized alanine (86.0% and 86.5%, respectively). At position 106, human sequences AA mainly utilized positively charged arginine (68.8%) and lysine (13.4%) and the arginine was found in 81.9% of the murine sequences. At position 115 of the C-terminal of VH CDR3, AA contained in human sequences were phenylalanine (49.7%), methionine (20.3%) and leucine (11.0%), as did the three most frequent amino acids in murine sequences (64.8%, 21.6% and 3.9%, respectively). At position 116, both human and murine sequences frequently utilized aspartic acid (84.2% and 76.6%, respectively). However, at position 117, the usage of tyrosine in mouse sharply exceeded the human (80.6% versus 32.4%).

### Junctional diversity

**Figure 4:**
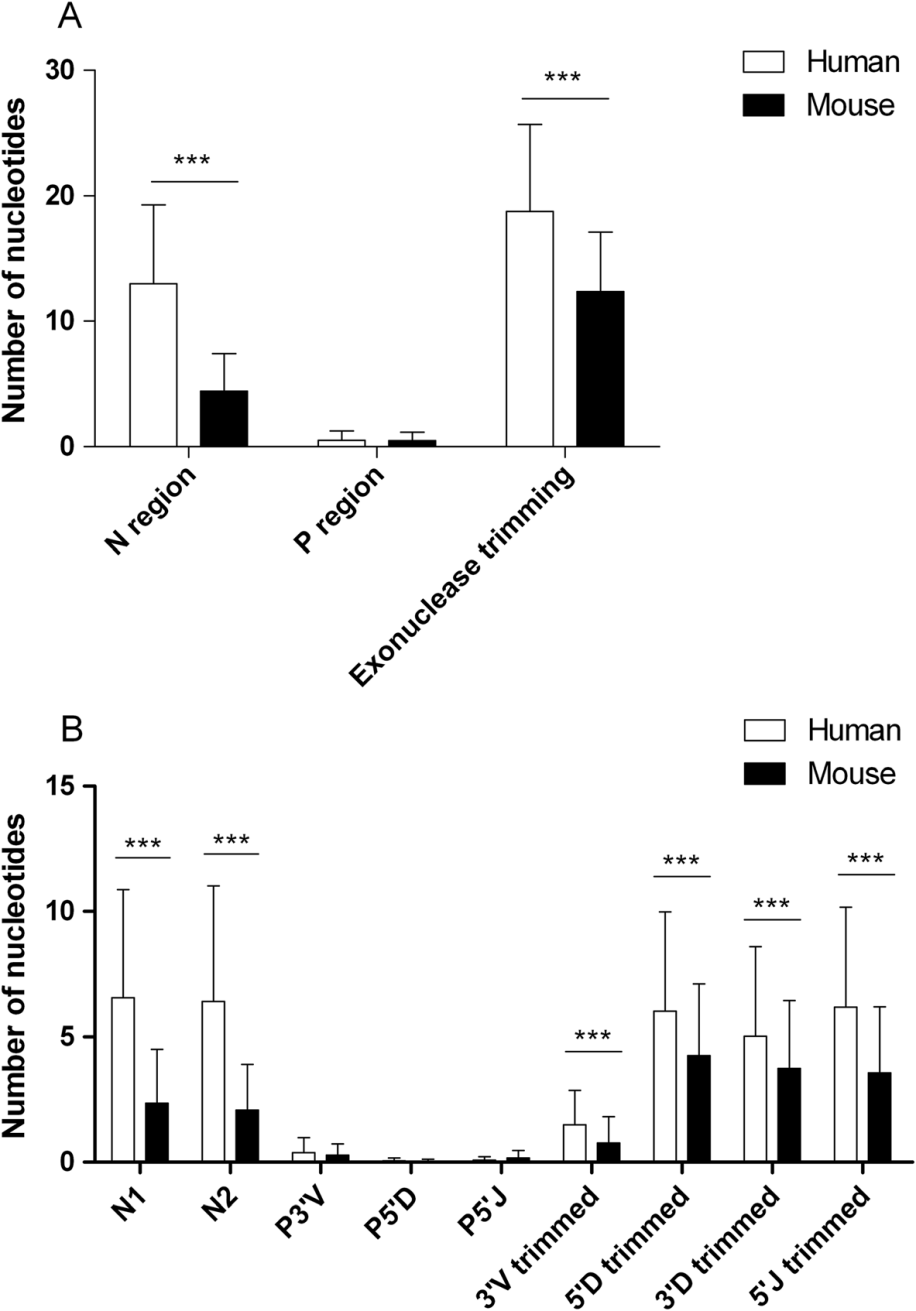
**The V-D-J junctional diversity created by P region, exonuclease trimming and N region addition significantly contributes to a large VH CDR3 repertoire. A** The numbers of N, P nucleotides added at the V-D-J junctions and nucleotides deleted by exonuclease trimming are shown, with the mean ± SD. The p values were determined using ANOVA. All statistically significant differences are indicated. * = p < 0.05, ** = p < 0.01, *** = p < 0.001. **B** The numbers of N1, N2, P3'V, P5'D and P5'J nucleotides added at the V-D-J junctions and nucleotides deleted at the 3'V, 5'D, 3'D and 5'J by exonuclease trimming are shown, with the mean ± SD. The p values were determined using ANOVA. All statistically significant differences are indicated. * = p < 0.05, ** = p < 0.01, *** = p < 0.001.

We analyzed all clearly identified 9,340 human sequences and 6,657 murine unique IG V-region sequences. The V-D-J junctional diversity results from the addition of palindromic “P” nucleotides (P region), exonuclease trimming and addition of “N” nucleotides (N region) at the V → D (N1) and D → J (N2) junctions (Figure 4A-B). Sequences occurring in the P-region (P3'V + P5'D + P5'J) addition accounted for 26.8% and 31.2% of overall sequences in human and mouse, respectively. There was a similar average number of nucleotides added in both human and murine P regions (0.5 ± 0.7 and 0.5 ± 0.7, respectively). The occurrence rate of exonuclease trimming was similar in human and mouse (99.9% and 99.4%, respectively). Sequences occurring in the N-region (N1 + N2) addition accounted for 98.2% and 83.1% of overall sequences in human and mouse, respectively. We found the average number of nucleotides added in the N region sharply greater in human than in mouse (13.0 ± 6.3 versus 4.4 ± 3.0, p < 0.001, ANOVA). For the average number of nucleotides lost due to exonuclease trimming, we found it sharply greater in human than in mouse (18.8 ± 7.0 versus 12.4 ± 4.8, p < 0.001, ANOVA). Figures for total addition and trimming in each domain are shown in Figure 4b and the numbers of nucleotides added in the N1 and N2 regions in human exceeded mouse (6.6 ± 4.3 versus 2.4 ± 2.2, 6.4 ± 4.6 versus 2.1 ± 1.8, respectively; each p < 0.001, ANOVA). The numbers of nucleotides trimmed in the 3'V region of human and mouse were less than those in the other three regions, but the number of nucleotides trimmed in each region was greater in human than in mouse (each p <0.001, ANOVA).

## Discussion

Human and mouse are the best developed models for the generation of IG diversity. However, due to limitations in the methodology, analysis of large data is difficult so that their IG information is not fully mining. Recently, IMGT/HighV-QUEST has been successfully applied in analysis of IG or TR repertoires. In the study, we used IMGT/HighV-QUEST to make comparative analysis of the human and mouse IG VHs from IMGT/LIGM-DB.

In the aspect of human and mouse germline genes, we noted significant preferential usage of several genes, i.e., IGHJ4 for humans and IGHV1-72, IGHD1-1, and IGHJ4 for mice. These IGHV genes are worth examining further.

Although presumably human and mouse must tolerate or eliminate similar classes of antigens and/or similar ranges of antigenic epitopes, their difference in AA composition of VH CDR3 was partly determined by the difference in junctional diversity and somatic hypermutation. The average number of nts added in the N region was sharply greater in human than in mouse (13.0 ± 6.3 versus 4.4 ± 3.0) with a higher occurrence rate of N-region addition than mouse (98.2% versus 83.1%). Hence, increased N-region addition results in more frameshift mutations that encode more amino acids and increase the diversity of human VH CDR3 by altering inserted positions and downstream codons. Thus, the AA composition of human VH CDR3 is more balanced. Scarce N-region addition in mouse might lead to shorter length and narrower distribution of VH CDR3. Moreover, fewer V region AA changes in human sequences occurred in low mutation regions (ranging from 0 to 5) (35.7% versus 70.6%) but more happened in high mutation regions (above 9) (49.9% versus 15.8%). Greater average numbers of AA changes in FR1, CDR1, FR2, CDR2 and FR3 suggests that a higher level of somatic mutation in human ensures immunoreaction to the diverse external environment.

Although analyses of human and murine IG repertoires showed significant differences, they also shared some similarities. Previous studies also identified that a bias usage of AA, such as tyrosine, alanine, aspartic acid, arginine, glycine and serine, occurred in both human and murine VH CDR3 [31,32]. Similar AA usage of human and murine VH CDR3 at conserved positions was observed except for murine tyrosine-rich position 117. A similar trend showing the mutation of V region was observed in both human and mouse and demonstrated that the number of sequences decreased as the number of AA changes increased. Moreover, a similar average number of nts added in the P region were observed in both human and mouse.

Previous studies reported that the generation mechanisms of Ig repertoires in different vertebrate groups were significantly different, which reflected different usage mechanisms [30]. Past studies confirmed similarity between the generation mechanisms for human and murine IG repertoire; however, their VH CDR3 AA compositions were significantly different. In this study, our results suggest that somatic hypermutation and junction diversity in human was significantly different from mouse, which reflected differences in number of AA changes due to somatic hypermutation and number of nucleotide insertion in N region. These findings may explain differences in their VH CDR3 AA compositions. Currently, the number of IG sequences of other species contained within the IMGT/LIGM-DB is still limited. Further sequencing for these species is necessary to improve knowledge about the generation of IG diversity. And IMGT/HighV-QUEST will strongly solve analysis of big data. Detection of IG repertoire has potential for clinical application; finally, we propose that IMGT/HighV-QUEST could be used to compare and analyze the high-throughput of large-scale sequence data before and after clinical treatment or vaccine research.

## Conclusion

In summary, we compared the compositions and characteristics of human and murine IG heavy chain V-regions from the IMGT/LIGM-DB through IMGT/HighV-QUEST. Our findings suggested that human had a higher number of AA changes due to somatic hypermutation and more abundant N-region addition than mouse, which might be an important factor leading to differences between human and mouse in the AA composition of VH CDR3. These findings are a benchmark for understanding VH repertoires and can be used to characterize the VH repertoire during immune responses. The study will allow standardized comparison for high throughput results obtained by IMGT/HighV-QUEST, the reference portal for NGS repertoire.

## Methods

### Retrieval of sequences

In December 2013, we queried human and murine sequences with Species (Homo sapiens (human) or Mus musculus (house mouse)), Molecule type (cDNA), Configuration type (rearranged), Functionality (productive) and Chain type (IG-Heavy) in the IMGT/LIGM-DB beta version (Version:1.0-rc2; Database release:LIGMDB_V12). From this search, we downloaded 10,507 human sequences and 8,362 murine sequences.

### IMGT/HighV-QUEST results for detailed statistical analysis

Overall sequences were entered into the IMGT/HighV-QUEST and two folders were obtained, each of which contained 11 text files: (1) Summary; (2) IMGT-gapped-nt-sequences; (3) Nt-sequences; (4) IMGT-gapped-AA-sequences; (5) AA-sequences; (6) Junction; (7) V-REGION-mutation-a nd-AA-change-table; (8) V-REGION-nt-mutation-statistics; (9) V-REGION-AA- changes-statistics; (10) V-REGION-mutation hotspots; (11) Parameters.

In text file (1), some human and murine sequences were filtered out, including “No results”, “Unknown functionality”, “Warnings”, and “more than 1” sequences. After these filters, we obtain 9833 human sequences (Additional file 2: Accession number for human IG VHs) and 7336 murine sequences (Additional file 3: Accession number for mouse IG VHs) with ‘1 copy’.

For human ‘1 copy’ sequences, we further remove 27 unproductive sequences, 6 ambiguous sequences (IMGT/JunctionAnalysis gives no results for this JUNCTION), 430 ambiguous or incomplete sequences (“FR-IMGT lengths” column or “CDR-IMGT lengths” column show “X” or “0”), and 30 ambiguous sequences (“AA JUNCTION” column show “X”), in turn. Similarly, for murine ‘1 copy’ sequences, we remove 11 unproductive sequences, 54 ambiguous sequences (IMGT/JunctionAnalysis gives no results for this JUNCTION), 478 ambiguous or incomplete sequences (“FR-IMGT lengths” column or “CDR-IMGT lengths” column show “X” or “0”), and 136 ambiguous sequences (“AA JUNCTION” column show “X”). After these filters, we obtain clearly identified and unique productive human (n = 9340) and murine (n = 6657) in-frame sequences for final research.

### IMGT/HighV-QUEST results statistical analysis

Through IMGT/HighV-QUEST, human (n = 9340) and murine (n = 6657) sequences were analyzed. From new text files (1), (6) and (9), we widely analyzed these sequences in Microsoft Office Excel, including germline gene usage, CDR3 length distribution and AA composition, analysis of the number of AA changes due to somatic hypermutation, and junctional diversity.

#### Germline gene usage

Gene usage frequencies in all human and murine V-D-J families were directly counted and computed in Excel (Additional file 1: Figure S1).

#### Analysis of the number of AA changes due to somatic hypermutation

AA changes in FR1-IMGT Nb, CDR1-IMGT Nb, FR2-IMGT Nb, CDR2-IMGT Nb and FR3-IMGT Nb were filtered from the text file (9) of human and mouse (number of AA changes of VH = FR1+ CDR1+ FR2+ CDR2+ FR3), and percentages of sequences of different numbers of AA changes and number of AA changes in each region were obtained (Figure 1A and B).

#### CDR3 length distribution and AA composition

In text file (1), AA JUNCTION was a CDR3 AA sequence with constant positions 104 and 108 which were deleted in analyzing the CDR3 sequences. Both human and murine CDR3 sequences (positions 105–117) were determined by IMGT numbering [26-28], and the CDR3 length distribution (Additional file 1: Figure S2) and frequencies of 20 different amino acids in CDR3 (Figure 2) were obtained; amino acid frequencies of all positions of overall CDR3 AA sequences were computed (Figure 3).

#### Junctional diversity

P3'V-nt nb, N1-REGION-nt nb, P5'D-nt nb, N2-REGION-nt nb, P5'J-nt nb, 3'V-REGION trimmed-nt nb, 5'D-REGION trimmed-nt nb, 3'D-REGION trimmed-nt nb and 5'J-REGION trimmed-nt nb were filtered from the text file (6) of human and mouse, and number of nucleotides inserted and deleted in each region was obtained (Figure 4).

### Software, statistics

IMGT/HighV-QUEST (version 1.2.0) was used for identification of sequences (VH, DH, JH and CDR3), evaluation of functionality and statistical analysis of sequence data; IMGT/V-QUEST (version 3.2.32) was used for identification of positions of CDR3; Microsoft Office Excel (version 2010) was used for storage, filtering and statistical calculation of sequences.

## Abbreviations

AA: Amino acid; nt: Nucleotide; CDR3: Complementarity determining region 3; IG: Immunoglobulin; IMGT: IMGT®, the international ImMunoGeneTics information system®; V: Variable; D: Diversity; J: Joining; IGHV: Immunoglobulin heavy variable; IGHD: Immunoglobulin heavy diversity; IGHJ: Immunoglobulin heavy joining; VH: Variable heavy; SHM: Somatic hypermutation.

## Competing interests

The authors declare no conflict of interest.

## Authors’ contributions

XY, LM, XH and BS designed research. XW, LZ and PW performed research. BS and LM analyzed data. BS wrote the paper. All authors read and approved the final manuscript.

## Supplementary Material

Additional file 1: Figure S1 Gene usage frequencies observed for the VH domain of human (n = 9340) and mouse (n = 6657) Ig sequenses published in the IMGT/LIGM-DB database. Each of the mapped and unmapped IGHV genes usage was calculated as the percentage of the total unique population of productive and in-frame sequences according to IMGT/HighV-QUEST Statistical Analysis Report. **A-H** IGHV subgroup (green bar), IGHD set (red bar), and IGHJ (yellow bar) gene utilization observed in human and mouse sequences. **Figure S2.** Length distribution of VH CDR3 regions (position 105-117) in all unique and productive human (n = 9340) and murine (n = 6657) sequences. Note that the length of the VH CDR3 region according to the definition used by IMGT. Click here for file

Additional file 2 Accession number for human IG VHs. Click here for file

Additional file 3 Accession number for mouse IG VHs. Click here for file
