# Supplementary material for: Comparative analysis of human and mouse immunoglobulin variable heavy regions from IMGT/LIGM-DB with IMGT/HighV-QUEST
Source: Theor Biol Med Model. 2014 Jul 3;11:30. doi: 10.1186/1742-4682-11-30 (PMC4085081; doi:10.1186/1742-4682-11-30)

**Fig. S1** Gene usage frequencies observed for the VH domain of human (n=9340) and mouse (n=6657) Ig sequenses published in the IMGT/LIGM-DB database. Each of the mapped and unmapped IGHV genes usage was calculated as the percentage of the total unique population of productive and in-frame sequences according to IMGT/HighV-QUEST Statistical Analysis Report. **A-H** IGHV subgroup (green bar), IGHD set (red bar), and IGHJ (yellow bar) gene utilization observed in human and mouse sequences.


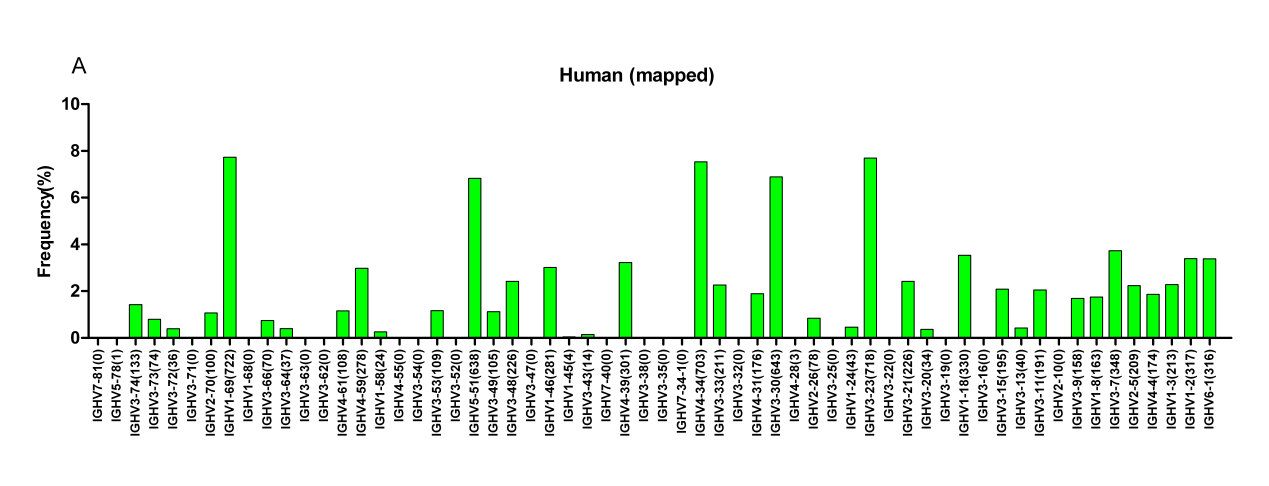


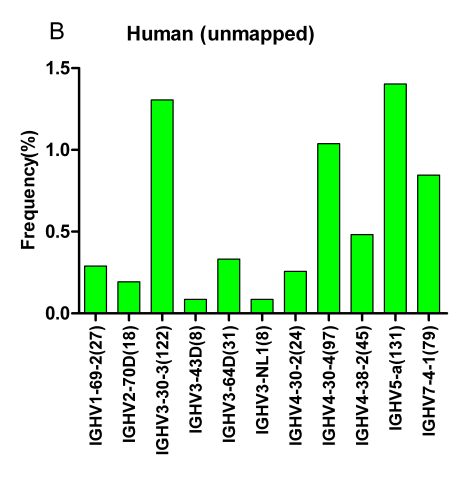

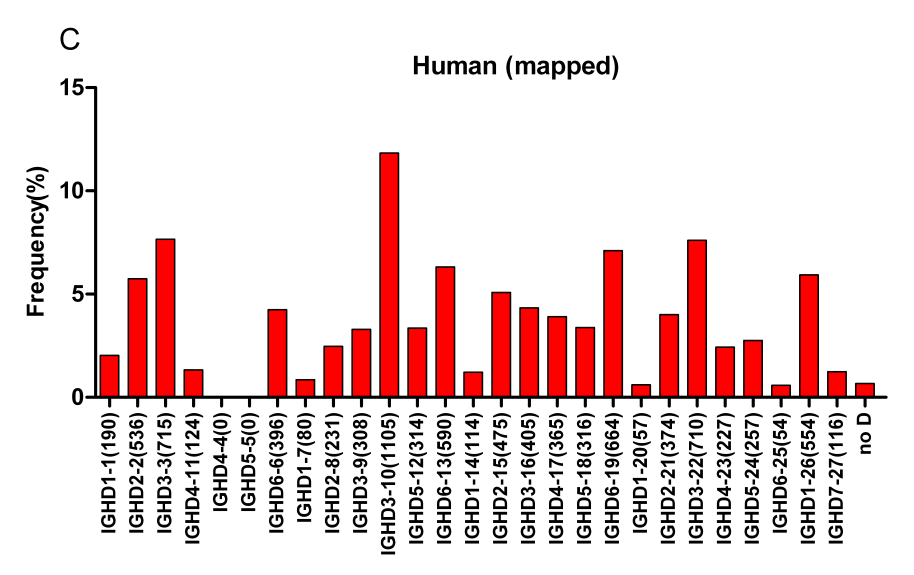

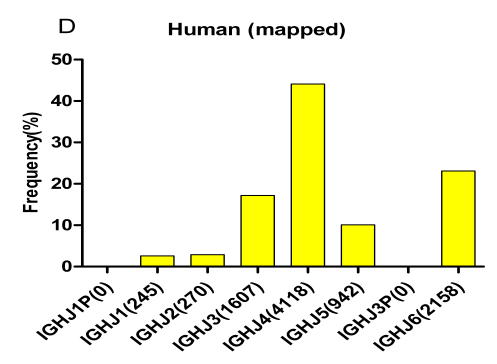


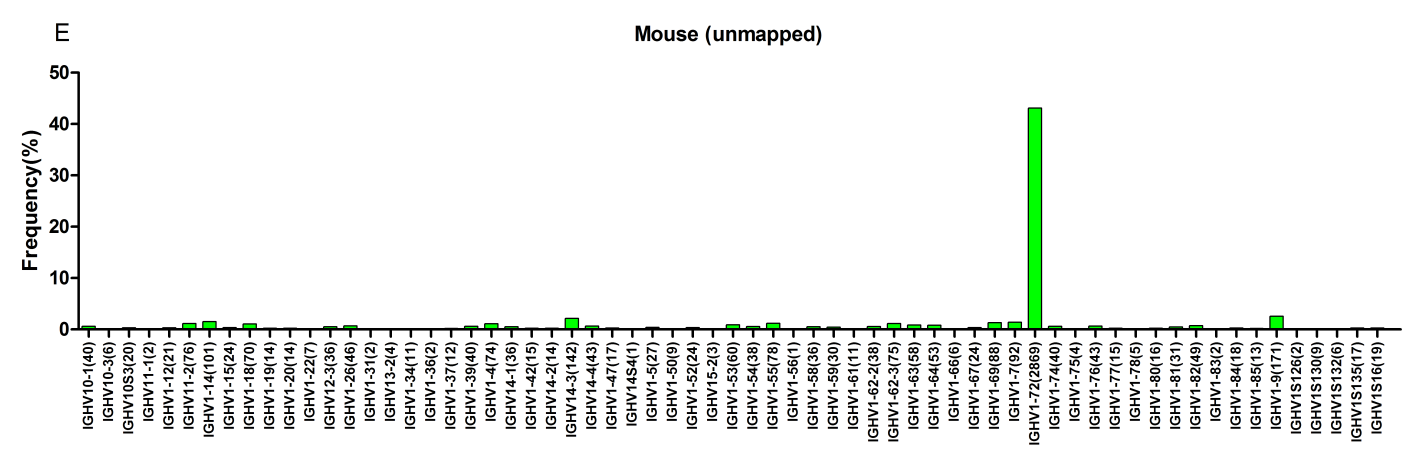


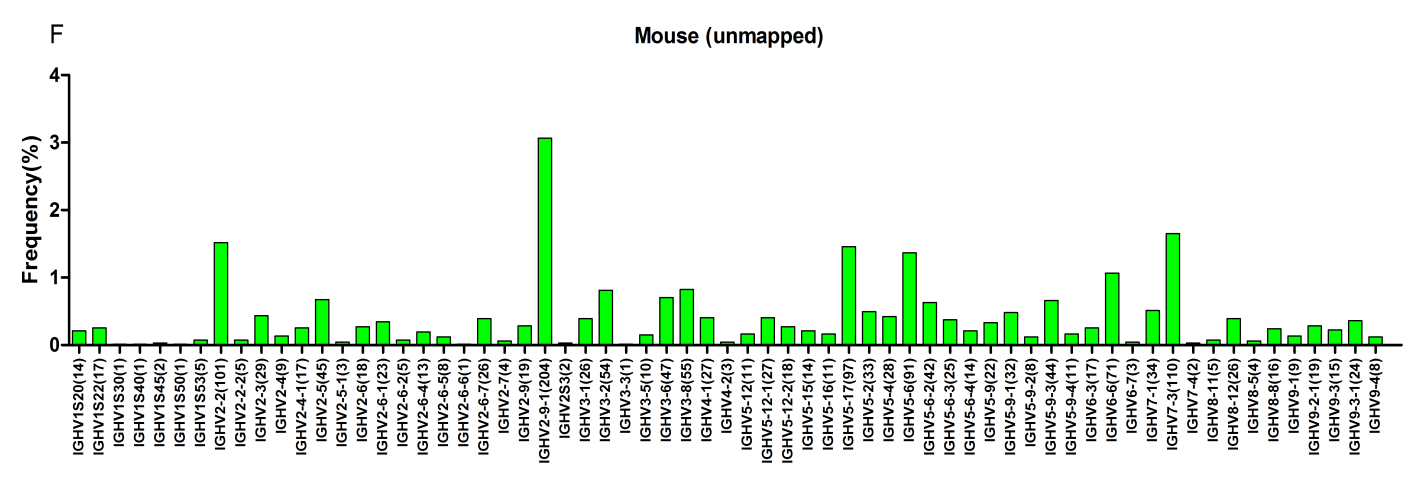


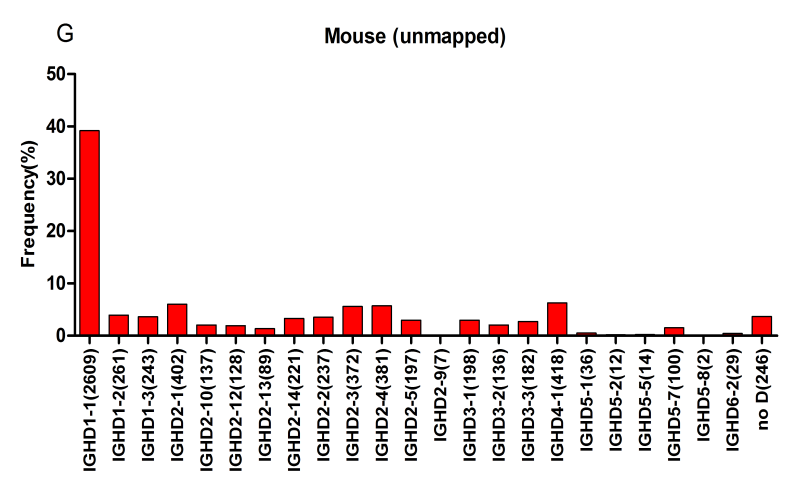

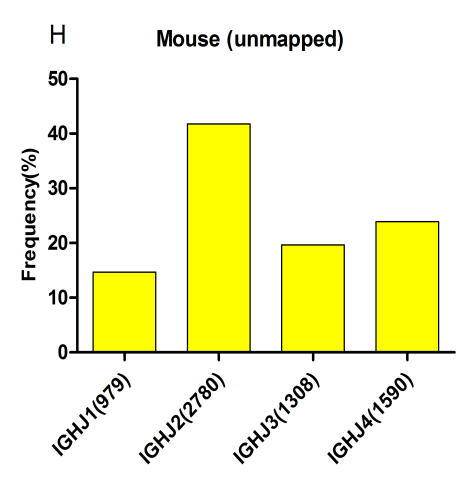


**Fig. S2** Length distribution of VH CDR3 regions (position 105-117) in all unique and productive human (n=9340) and murine (n=6657) sequences. Note that the length of the VH CDR3 region according to the definition used by IMGT.


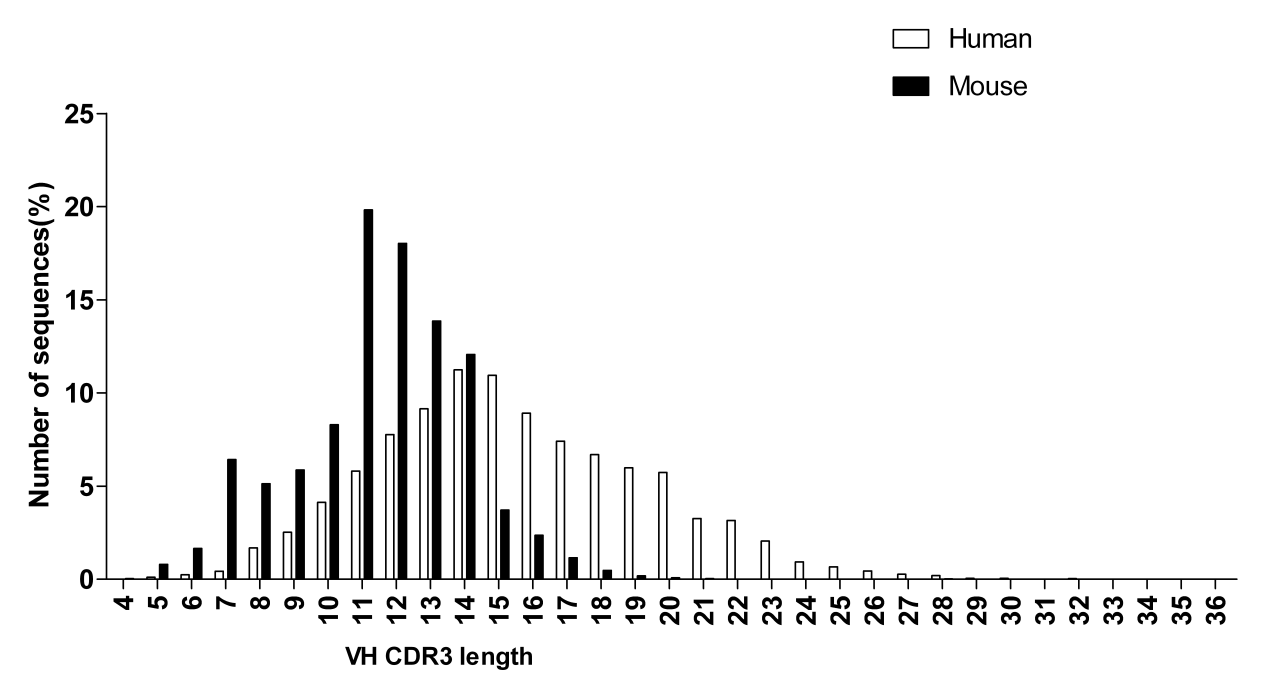

Supplement: Additional file 1: Figure S1 — Gene usage frequencies observed for the VH domain of human (n = 9340) and mouse (n = 6657) Ig sequenses published in the IMGT/LIGM-DB database. Each of the mapped and unmapped IGHV genes usage was calculated as the percentage of the total unique population of productive and in-frame sequences according to IMGT/HighV-QUEST Statistical Analysis Report. A-H IGHV subgroup (green bar), IGHD set (red bar), and IGHJ (yellow bar) gene utilization observed in human and mouse sequences. Figure S2. Length distribution of VH CDR3 regions (position 105-117) in all unique and productive human (n = 9340) and murine (n = 6657) sequences. Note that the length of the VH CDR3 region according to the definition used by IMGT. [file 1742-4682-11-30-S1.docx]
